# Ensartinib targeted conversion surgery for ALK-positive unresectable locally advanced non-small cell lung cancer: a case report

**DOI:** 10.1186/s13019-026-03940-1

**Published:** 2026-03-03

**Authors:** Sijin Huang, Chuan Yuan, Yanheng Wang, Binbin Chen, Wenfang Tang, Haiming Jiang, Wenhao Li, Yi Liang, Weizhao Huang

**Affiliations:** 1 Guangdong Medical University, Zhanjiang, 524023 Guangdong China; 2 Department of Cardiothoracic Surgery, Zhongshan City People’s Hospital, Zhongshan City, Guangdong Province China; 3 Institution of Advanced Diagnostic and Clinical Medicine, Zhongshan City People’s Hospital, Zhongshan City, Guangdong Province China; 4 Zhongshan Clinical Medicine Research Institute, Zhongshan City, Guangdong Province China

**Keywords:** Targeted conversion surgery, Anaplastic lymphoma kinase, Non-small cell lung cancer, Ensartinib, Pathological complete response, Neoadjuvant therapy

## Abstract

Currently, there is limited evidence supporting ensartinib’s efficacy as a neoadjuvant treatment for locally advanced anaplastic lymphoma kinase (ALK)-positive non–small cell lung cancer (NSCLC). This case study describes a 65-year-old female who presented with an incidental pulmonary lesion and was diagnosed with unresectable locally advanced stage IIIB (cT3N2M0) ALK-positive NSCLC. After multidisciplinary evaluation, she underwent 12 weeks of neoadjuvant ensartinib therapy (225 mg/day, later reduced to 175 mg/day), achieving a 78.4% reduction in tumor volume and reassessment as resectable. Subsequently, video-assisted thoracoscopic surgery (VATS) comprising a right upper lobectomy and systematic lymph node dissection resulted in R0 resection. Postoperative pathological analysis confirmed pathological complete response (pCR). During an fifteen-month follow-up, no recurrence of the disease was detected.

These results indicate that ensartinib may provide potential benefit for patients with locally advanced ALK-positive NSCLC by optimizing surgical feasibility and survival outcomes.

**Supplementary Information:**

The online version contains supplementary material available at 10.1186/s13019-026-03940-1.

## Introduction

As critical driver mutations in non–small cell lung cancer (NSCLC), anaplastic lymphoma kinase (ALK) gene rearrangements account for 3%–7% of all cases [1]. Although ALK tyrosine kinase inhibitors (TKIs) significantly improve survival outcomes in advanced-stage patients, treatment strategies for locally advanced disease remain controversial. Standard guidelines recommend neoadjuvant chemotherapy or chemoimmunotherapy [2]; however, there is insufficient evidence supporting the use of ALK-TKIs in this setting. Neoadjuvant therapy is aimed at tumor burden reduction, disease downstaging, and improved radical resection outcomes. Nevertheless, two clinical trials assessing neoadjuvant chemoimmunotherapy in patients with ALK and epidermal growth factor receptor (EGFR) mutations reported low pathological complete response (pCR) rates of 17.2% [3] and 18.1% [4], respectively, with toxicities and.

tumor progression frequently making surgery impossible. Owing to limited high-level evidence, for ALK-positive NSCLC, the choice between neoadjuvant chemoimmunotherapy and targeted therapy remains contentious. Ensartinib, a second-generation ALK-TKI, shows strong penetration of the blood–brain barrier (intracranial response rate: 63.6%) and manageable adverse effects (mainly grade 1–2 rashes and transaminase elevation) [5]. Its role in advanced.

ALK-positive NSCLC includes an objective response rate (ORR) of 75% and a median progression-free survival (mPFS) of 41.5 months [6], indicating its potential role in neoadjuvant therapy.

Conversion surgery refers to surgical resection after systemic therapy in initially unresectable locally advanced disease, distinguishing it from salvage surgery (performed after disease progression) or planned induction therapy. This report describes a case of unresectable stage IIIB ALK-positive NSCLC treated with neoadjuvant ensartinib, resulting in pCR.

**Fig. 1:**
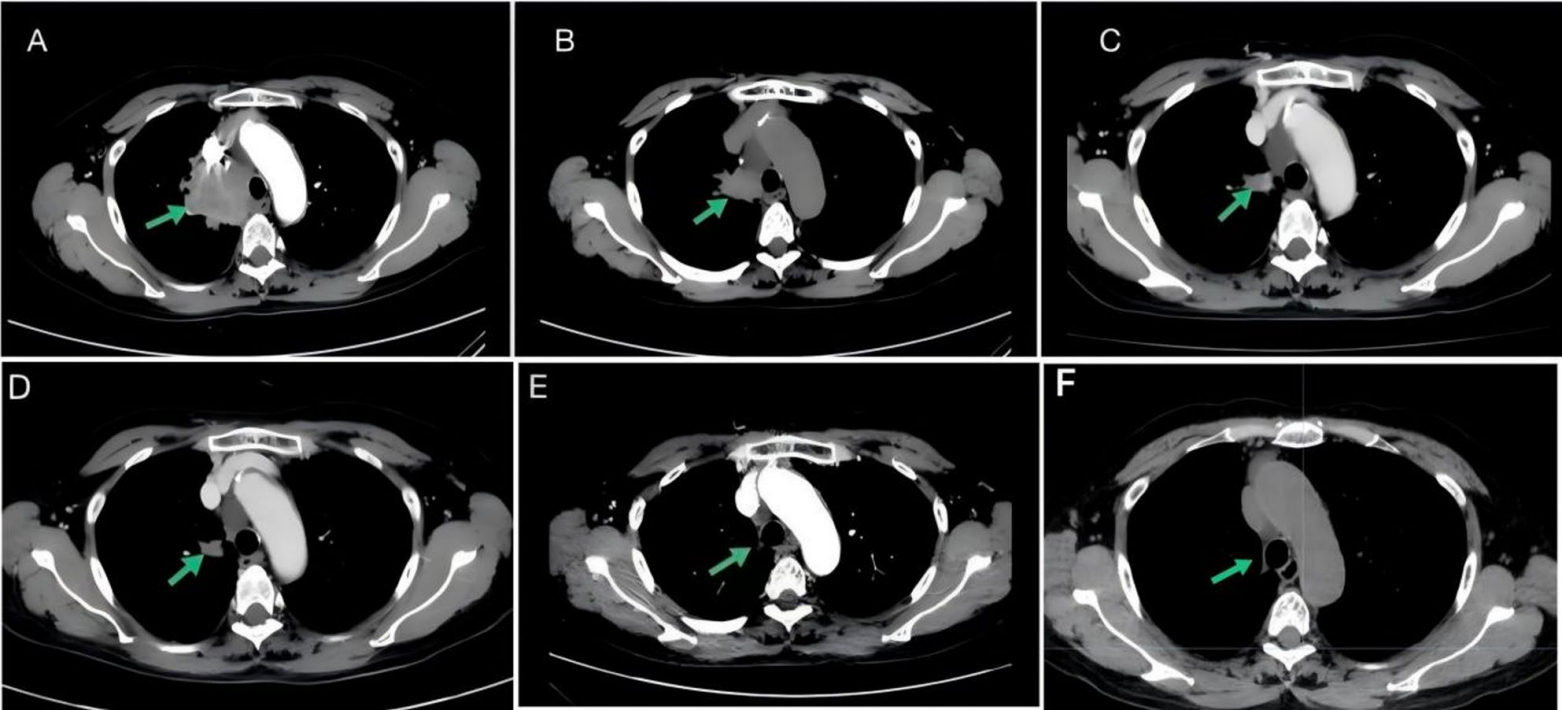
(**A**) Contrast-enhanced chest CT (May 9, 2024): A 55.5 × 43.5 mm soft-tissue mass adjacent to the right hilum with mediastinal lymphadenopathy (no distant metastasis). (**B**) Two weeks post-ensartinib (May 31, 2024): Primary lesion reduced to 32 × 24 mm; mediastinal lymph nodes regressed. (**C**) Week 8 (July 17, 2024): Lesion further reduced to 20 × 12 mm. (**D**) Week 12 (August 20, 2024): Residual lesion measured 12 × 11 mm (78.4% reduction from baseline). (**E**) Postoperative CT (April 10, 2025): No recurrence. (**F**) Postoperative CT (November 10, 2025): No recurrence

## Case presentation

**Fig. 2:**
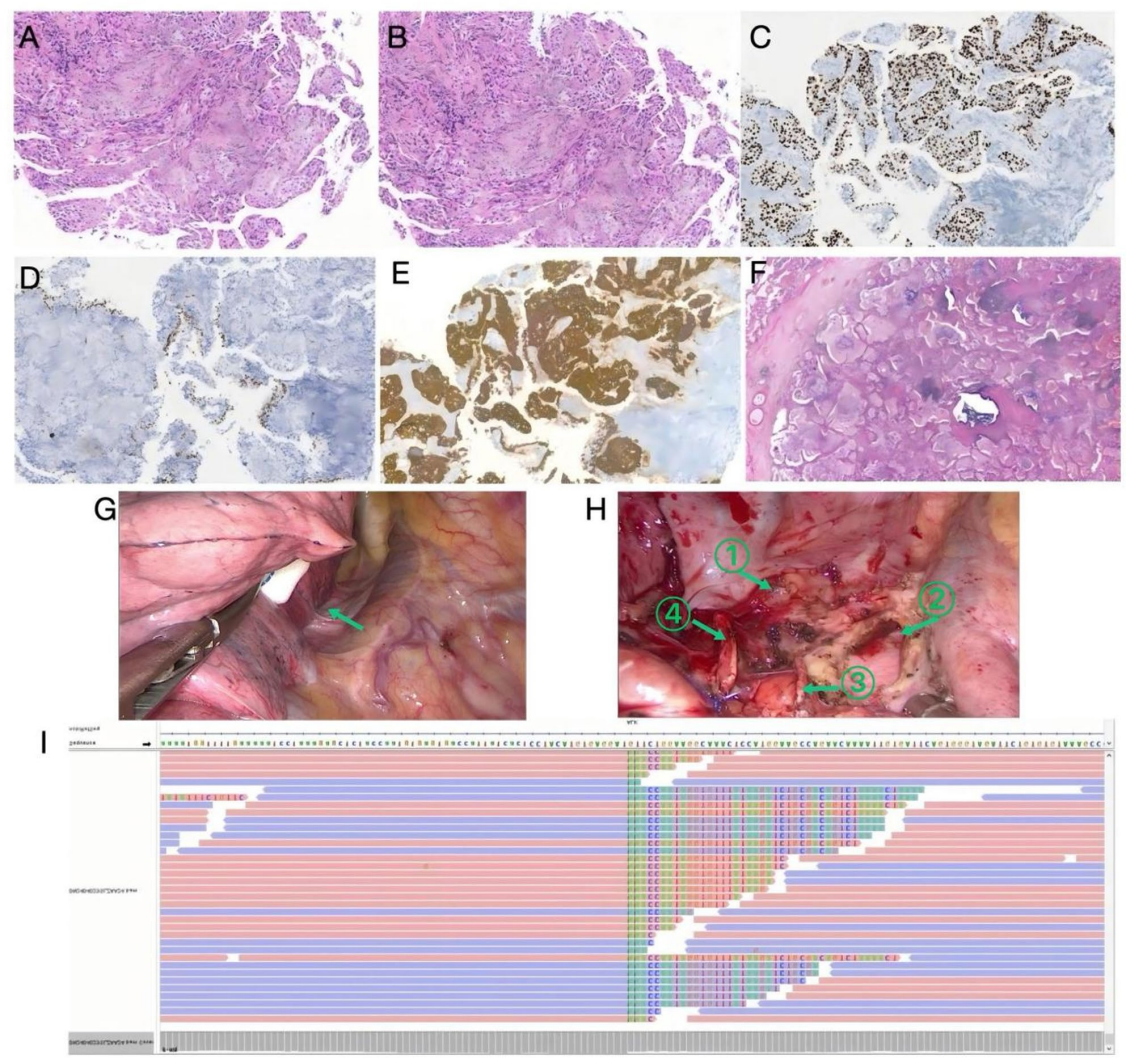
(**A–B**) Pretreatment H&E staining: Squamous carcinoma with nested growth. (**C**) Pretreatment P40 (+). (**D**) Pretreatment TTF-1 (−). (**E**) Pretreatment CK5/6 (+). (**F**) Posttreatment H&E: Lymphocytic and histiocytic infiltration without residual tumor. (**G**) Intraoperative exploration revealed a tumor located in the right upper lobe, firmly adherent to the azygos vein and the right upper mediastinal pleura. (**H**) En bloc resection of the right upper lobe and azygos vein (arrow ①) was performed. As the pericardium was involved, the right upper mediastinal lymph nodes were dissected along with the overlying pericardium (arrow ②). The stumps of the superior trunk of the pulmonary artery (arrow ③) and the upper lobe bronchus (arrow ④) are shown. (**I**) NGS: ALK fusion mutation (V3a)

A 65-year-old female was hospitalized with right-sided chest pain in May 2024. Bronchoscopic biopsy from the lesion at the apical segment (S1) of the right upper lobe bronchus confirmed squamous cell carcinoma (Figs. 2A–B). Immunohistochemistry (Figs. 2 C–E) demonstrated PD-L1 (clone 22C3) positivity (tumor proportion score [TPS] ~ 90%), INI-1 (+), CK5/6 (+), P40 (+), and Ki-67 (+). Next-generation sequencing (NGS) was performed due to high PD-L1 expression and advanced stage, which revealed an ALK rearrangement (Fig. 2I), confirming stage IIIB (cT3N2M0, 8th edition TNM classification) NSCLC. With a multidisciplinary evaluation affirming the tumor’s unresectability, neoadjuvant therapy with ensartinib (225 mg/day) was initiated on May 17, 2024. The patient developed grade 1–2 rash and elevated transaminases, necessitating a dose reduction to 175 mg/day, which was well tolerated. After 12 weeks of therapy, the tumor volume decreased by 78.4%, resulting in a partial response (Figs. 1A–D).

**Fig. 3:**
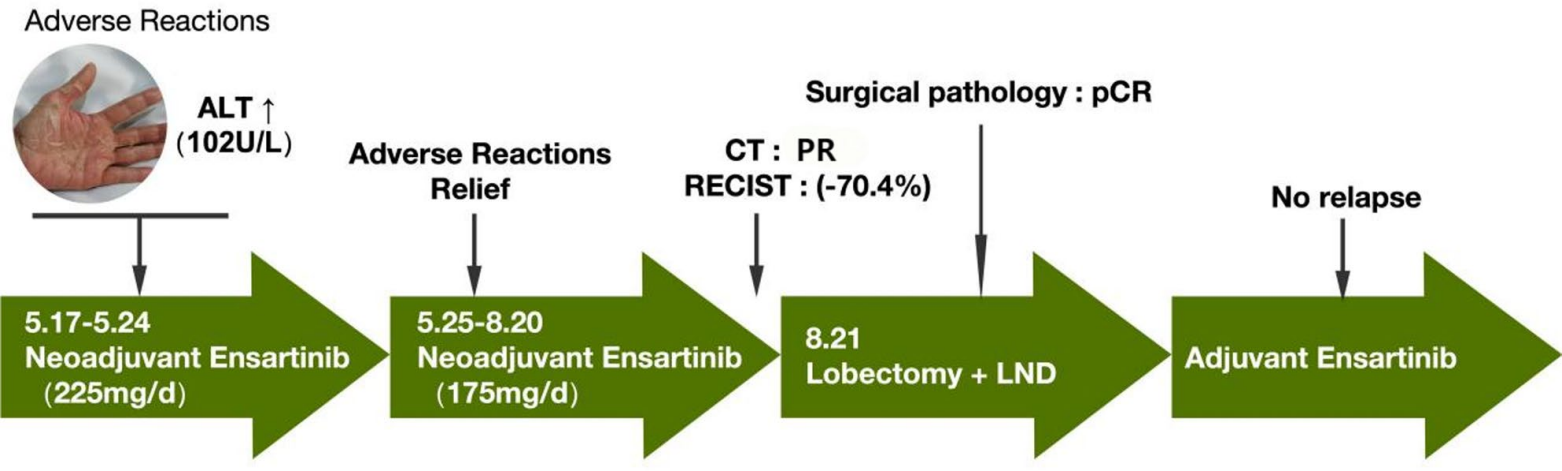
Treatment timeline, including RECIST (Response Evaluation Criteria in Solid Tumors), PR (partial response), pCR (pathological complete response), LND (lymph node dissection)

The tumor was then re-evaluated as resectable, and consequently, the patient underwent video-assisted thoracoscopic surgery (VATS) right upper lobectomy with systematic lymph node dissection including stations 2R, 4R, 7, 10R, 11, 13 and 14 on August 21, 2024 (Figs. 2G-H). Intraoperatively, the tumor bed and lymph nodes were considerably reduced. Postoperative pathological analysis detected no residual tumor cells in the tumor bed, lymph nodes, surgical margins, or bronchial stump, confirming pCR (Fig. 2F) and ypT0N0M0 (pathological complete response). Adjuvant ensartinib therapy (225 mg/day) was continued. Follow-up chest CT scans over 8 months(Fig. 1E) and 15 months(Fig. 1F) revealed no recurrence. The treatment timeline is shown in Fig. 3.

## Discussion

Being highly heterogeneous, stage III NSCLC requires therapeutic strategies that are tailored to molecular profiles and clinical staging. This patient, diagnosed with stage IIIB squamous carcinoma, ALK fusion, and PD-L1 high expression (TPS 90%), had to choose between chemoimmunotherapy and targeted therapy. The KEYNOTE-671 trial [7] showed that the combination of perioperative pembrolizumab and chemotherapy improved overall survival (OS) and event-free survival (EFS) in resectable stage II–IIIB NSCLC. However, subgroup analyses demonstrated that patients who are ALK/EGFR-positive derive limited benefit, signifying that immunotherapy may not be very effective in this population due to factors such as immunosuppressive microenvironments and insufficient T-cell infiltration.

Although ALK rearrangements are predominantly found in adenocarcinoma, rare cases have been reported in squamous cell carcinoma, often suggesting adenosquamous differentiation or immunohistochemical overlap. In our case, despite squamous markers (P40+, CK5/6+), the presence of ALK fusion suggests a possible adenosquamous component or rare true squamous ALK-positive subtype, underscoring the importance of comprehensive molecular profiling in advanced NSCLC regardless of histology.

For ALK-positive NSCLC, targeted therapy in the perioperative setting is attracting increasing interest. For example, the SAKULA trial [8] reported a pCR rate of 29% with neoadjuvant therapy with ceritinib, while alectinib achieved a pCR rate of 37.5% in patients who were in stages IIIA–IIIB. Ensartinib, which is a second-generation ALK-TKI, competitively inhibits ALK kinase activity, suppresses downstream signaling, and induces apoptosis [9]. Compared to first-generation TKIs such as crizotinib, ensartinib enhances central nervous system penetration and activity against resistance mutations [10]. Additionally, ensartinib’s favorable safety profile allows for dose adjustments to manage primary adverse effects, including grade 1–2 rashes and transaminase elevation. However, evidence favoring its role in neoadjuvant therapy for locally advanced NSCLC remains scarce—currently, only case reports and small retrospective studies are available. Indeed, this case showed that targeted induction therapy with ensartinib successfully achieved the expected therapeutic effects of neoadjuvant treatment.

During therapy, the patient developed palmar rashes and elevated alanine aminotransferase (ALT), which were managed by reducing the dose from 225 mg/day to 175 mg/day. Consequently, she successfully completed 12 weeks of preoperative neoadjuvant therapy and underwent R0 resection. Postoperative pathology confirmed pCR, further emphasizing the potential of ensartinib as a neoadjuvant treatment for locally advanced NSCLC. Despite pCR, adjuvant ensartinib was continued to reduce recurrence risk, given the initial advanced stage and potential for micrometastatic disease.

The choice of a minimally invasive approach (VATS) was considered feasible after significant tumor downstaging and regression, consistent with evolving practices in thoracic surgery following effective neoadjuvant therapy.

### Limitations

This study has several limitations. First, it is a single-case report, which limits generalizability. Second, the follow-up period (15 months) is relatively short to assess long-term outcomes and durability of response. Third, no comparative analysis with chemoimmunotherapy was performed. Future prospective studies with larger cohorts and longer follow-up are needed to validate these findings.

## Conclusion

Despite scarce evidence, ensartinib shows considerable neoadjuvant therapy efficacy in patients with ALK-positive NSCLC. By optimizing surgical feasibility and improving survival outcomes, it may provide potential benefit for those with locally advanced disease, thus providing new insights into the therapeutic paradigm for this patient population.

## Electronic Supplementary Material

Below is the link to the electronic supplementary material.

Supplementary Material 1

## Data Availability

No datasets were generated or analysed during the current study.

## Declarations

Additional Video.mp4: VATS procedure demonstrating R0 resection (Video consent obtained, de-identified)
